# Relative Health Effects of Education, Socioeconomic Status and Domestic Gender Inequity in Sweden: A Cohort Study

**DOI:** 10.1371/journal.pone.0021722

**Published:** 2011-06-29

**Authors:** Susan P. Phillips, Anne Hammarström

**Affiliations:** 1 School of Medicine, Queen's University, Kingston, Ontario, Canada; 2 Department of Public Health and Clinical Medicine, Umeå Centre for Gender Studies in Medicine, Umeå University, Umeå, Sweden; Genentech Inc., United States of America

## Abstract

**Introduction:**

Limited existing research on gender inequities suggests that for men workplace atmosphere shapes wellbeing while women are less susceptible to socioeconomic or work status but vulnerable to home inequities.

**Methods:**

Using the 2007 Northern Swedish Cohort (n = 773) we identified relative contributions of perceived gender inequities in relationships, financial strain, and education to self-reported health to determine whether controlling for sex, examining interactions between sex and other social variables, or sex-disaggregating data yielded most information about sex differences.

**Results and Discussion:**

Men had lower education but also less financial strain, and experienced less gender inequity. Overall, low education and financial strain detracted from health. However, sex-disaggregated data showed this to be true for women, whereas for men only gender inequity at home affected health. In the relatively egalitarian Swedish environment where women more readily enter all work arenas and men often provide parenting, traditional primacy of the home environment (for women) and the work environment (for men) in shaping health is reversing such that perceived domestic gender inequity has a significant health impact on men, while for women only education and financial strain are contributory. These outcomes were identified only when data were sex-disaggregated.

## Introduction

An ever-increasing volume of evidence documents that external social conditions such as socio-economic inequality affect individual health to ultimately increase morbidity and shorten life. Although genetic makeup may dictate one's basic endowment of individual resources, social entitlements and deprivations likely act as switches that turn on or off the body's ability to maximize those inherent assets.

Most widely studied of these switches has been the association between socio-economic status (SES) or wealth, and health. SES may be measured at the individual level by examining relative income, educational attainment, occupational class, or deprivation.[1] It can also be examined across groupings as large as nations by assessing income inequality, that is, the gap between the percent of the population and the percent of that population's earnings relative to the whole.[2] Regardless of the measure, in general, lower SES brings greater morbidity and mortality.

We could find no studies of SES and overall health that included a measure of perceived gender equity as an independent explanatory variable. By gender equity we mean fairness and justice in the distribution of benefits and responsibilities between women and men. The concept recognizes that women and men have different needs and power and that these differences should be identified and addressed in a manner that rectifies the imbalance between the sexes. In studying whether the effects of SES are modified by the constraints of race and ethnicity in the U.K. Cooper was able to indirectly hypothesize that gender inequity is bad for the health of some women.[3] She examined whether the links between SES and self-reported health varied by sex, finding that they did not among white men and women, but for ethnic groups, particularly those from Pakistan and Bangladesh, being female added to the disadvantage of economic deprivation. Although no explicit measure of gender inequity was used in this study the variation observed could indicate that greater acceptance of gender inequality, that is, of sex disparities in rights, decision-making, or access to and control of resources, at the group level may have detrimental health effects on women, effects that intersect with and magnify those of SES, alone. Socio-economic and gender inequities are thought to intersect, interact, and possibly confound each other, however research has primarily examined the effects of disparities across but not within households.[4] In this study we explore the independent and relative impact of socio-economic and perceived household gender inequities on self-reported health outcomes, testing various measures and models.

Initial key research on SES and health was blind to sex differences.[5] Subsequent enquiry suggested that not all measures of SES have a common meaning for men and women. While SES, alone, is generally more closely linked to health outcomes in men than women, measures of lack of material resources and relative deprivation tend to show a linear, inverse relationship with health for both sexes.[1], [6], [7] The combined SES of household members may more accurately reflect lived economic status than do individual measures. Similarly, in settings where male incomes exceed female, for women who have a male partner, his occupational status or the occupational class that is dominant within the household can be a better predictor of health outcomes than is her occupation.[3], [8], [9] For men with female partners, occupational status of that partner generally does not explain male health status. The difference in observed effect depending on sex means that use of individual occupational status as a measure of SES may have different meanings for men and women and can, therefore, be problematic. Equally challenging is the meaning of individual educational attainment or individual income. Although these may measure SES in men and single women, the contribution of partners' incomes and educational status has a significant bearing on the SES of women who co-habit with male mates.[8] Measures of relative deprivation, that is, of inability to afford those goods and activities that are typical of a specific society at a given time, appear to maximize accuracy and minimize gender bias without necessitating stratification of research data by marital status.[10]

Dissecting the differences between women and men in the relationship between relative wealth and health may foster greater understanding of how social determinants translate into individual health outcomes. Within the past two decades social epidemiologists have begun to focus on how the health effects of SES vary by sex. In men the observed association between occupational class and health appears to be mediated by psychosocial as well as physical conditions in the workplace, and by job security.[11] However, among women, but not men, domestic workload and perceived control at home have a significant impact on health, while both sexes are disadvantaged by household material deprivation.[12] In general, it would appear that a sense of relative deprivation has a negative impact on health regardless of gender. For men individual SES, intertwined with workplace status and control seem central, whereas for women household SES and individual control or equity at home may be key determinants of health.

Informed by the literature reviewed above, our study will use a variety of measures of socio-economic status, including a deprivation scale, to explore relative contributions of these to self reported health of men and women. Unlike the above literature, the relative contribution of perceived gender equity will be identified, not solely by determining whether the relationship of interest is different for women and men but also by including an explicit measure of gender equity among the independent variables.

## Methods

### Participants

The Northern Swedish Cohort includes all pupils who in 1981 attended the last year of compulsory school (age 16) in a medium-sized industrial town in the north of Sweden. At the 26-year follow-up 93.9% (n  = 1006) of those still alive of the original cohort (n = 1083) continued to participate. For this study all participants who were cohabiting or married at the time of the most recent (2007) follow-up (n = 773) were included.

All participants were surveyed at ages 16, 18, 21, 30 and 42 with a comprehensive questionnaire linked to register data. Data were collected by group questionnaires at ages 16 and 18. At ages 21, 30 and 40 participants were invited to reunions with former classmates. Those who could not attend (and those at age 18 who had finished school) received a mailed questionnaire. If data were missing, participants were contacted by phone for supplementary information. More detailed descriptions of the method have been published elsewhere.[13], [14] For this study, the 2007 follow-up at age 42 was used.

All questionnaires included about 90 questions regarding family background, work experience, work environment, financial position, social support, civil status, domestic work, health situation etc (see Appendix S1). The questionnaire was derived from well-known and validated sources such as the Swedish national survey of living conditions [15] and the Low-Income Study.[16]

The study, including consent methodology, has ethics approval from the Ethics Committees of Uppsala University, Umeå University and Statistics Sweden as well as by the Regional Ethics Vetting Board in Umeå. Written consent has not been requested from these committees. The respondent is regarded as giving written consent when answering the questionnaire. Participants were/are able to opt out at any time simply by not completing any of the waves of the survey.

Health outcomes at age 42 were measured by asking for self-rated health measured as good = 0, poor, or something in between good and poor = 1.[17]

Four independent variables were included in the complete model. Low education at age 42 was measured with one question. Those with university exam were defined as high-educated (36.6%) while those with upper secondary school education or less were defined as low-educated (63.4%). Sex/gender was coded as woman = 1, man = 2. Lack of Financial strain/relative deprivation at age 42 was measured as an index based on 11 questions as to whether respondents had been forced to do without any of the following during the last twelve months: cooked meal, buying clothes they or the family needed, paying bills on time, going to the cinema/concert/theatre, inviting friends home, travelling to see relatives or friends, buying presents, going on vacation, subscribing to a newspaper, spending time on hobbies or leisure activities, going to restaurants/pubs.[18] Each question was based on a four point Likert scale with the answer alternatives of often = 0, seldom = 1, never = 2, non applicable = 3. As some participants misunderstood the last alternative, the answer alternatives ‘never’ and ‘not applicable’ were merged into alternative  = 2. Thus, the scale of the index was 0–22. Overall assessments of perceived gender inequity in the couple relationship at age 42 were assessed by asking, “How gender equal do you consider your couple relationship to be?”[19] The question had a 5 point Likert-type scale with options of “totally gender equal” ( = 1), “quite gender equal”, “somewhat gender equal”, “not especially gender equal”, and “not gender equal at all” ( = 5).

To identify reverse causation earlier health status that could influence education and gender equity in one's relationship has been considered. Ideally, earlier health status would be indicated via self-reported health asked at baseline (age16) when the entire cohort had the same education and before the ages of marriage/partnership. However, as the question of self-rated health was not asked at that time, we used a composite of recorded measures of somatic and psychological symptoms at age 16 as a proxy for self-rated health.[17] This index was constructed from 21 different somatic symptoms measured on three-point Likert scales - from 0 (no problems) to 2 (serious problems) (range 0–42) - and frequency of nervousness or depressive symptoms (never  =  0 to often  =  3). All questions addressed symptoms in the preceding twelve months, eg musculoskeletal disorders, gastric complaints, allergic problems, headache, tiredness, dizziness, overstrain, infections, accidental injuries.

SPSS18.0 was used for data analysis. A p-value <0.05 or a 95% confidence interval for ORs was chosen as statistically significant. To test significance chi-square was used for dichotomous variables and t-test for continuous variables. Multivariate logistic regression analyses were used to estimate the odds ratios (OR) with 95% confidence intervals (CI) for health outcomes in relation to the independent variables, after controlling for reverse causation (i.e. earlier health status). The logistic regression models were tested for accuracy with tests of model chi-square, which indicated a moderate fit. Multiplicative interaction analyses were performed between independent variables.

## Results

Table 1 shows that men had a lower education level than women, but also experienced less financial deprivation and less gender inequity. No significant differences between men and women were found for current suboptimal self-rated health. At age 16, girls had had more somatic, depressive and nervous symptoms than boys.

**Table 1:** Distribution of variables used in the analyses among men and women (per cent, means and standard deviation).

	Men	Women	p
Suboptimal self-rated health at age 42 -%	29.9	32.6	0.438^a^
Low education at age 42- %	66.8	56.9	0.005^a^
Somatic complaints at age 16 - means (standard deviation) (range 0–42)	6.44 (4.26)	7.39 (3.97)	0.002^b^
Frequency of depressive symptoms at age 16 - means (standard deviation) (range 0–3)	0.59 (0.53)	0.98 (0.52)	< 0.001^b^
Frequency of nervous symptoms at age 16 - means (standard deviation) (range 0–3)	0.18 (0.40)	0.39 (0.54)	< 0.001^b^
Lack of financial strain at age 42 - means (standard deviation) (range 0–22)	20.11 (3.74)	18.85 (4.75)	< 0.001^b^
Perceived gender inequity at age 42- means (standard deviation) (range 1–5)	1.78 (0.82)	1.95 (0.94)	0.007^b^

a Chi square,

b T-test.

Table 2 examines the associations between various independent variables using different models in a logistic regression with suboptimal self-rated health at age 42 as the outcome.

**Table 2:** Logistic regression analyses for suboptimal self-rated health at age 42.

	Model 0		Model 1		Model 2		Model 3		Model 4^a^	
	OR	CI	OR	CI	OR	CI	OR	CI	OR	CI
Low education	1.49	1.08–2.06			1.49	1.08–2.06	1.46	1.01–1.94	1.51	1.08–2.12
Lack of financial strain	0.94	0.90–0.97					0.94	0.91–0.98	0.95	0.92–0.99
Sex/gender	0.88	0.65–1.20	0.90	0.66–1.23	0.87	0.64–1.18	0.96	0.70–1.32	1.05	0.70–1.43
Gender inequity	1.20	1.01–1.42	1.19	1.01–1.41	1.19	1.01–1.41	1.15	0.70–1.37	1.14	0.92–1.37

a after control for reversed causation.

Model 0 = crude OR, model 1 = control for sex/gender and perceived gender inequity, model 2 = model 1+low education, model 3 = model 2+financial deprivation, model 4 = model 3 after control for reversed causation.

Bi-variate correlations for each independent variable, except sex/gender, with suboptimal self-rated health at age 42 are statistically significant. Low education and lack of financial strain remain significant in all models. Perceived gender inequity becomes insignificant when financial strain is added to the model (model 3). Controlling for the effect of poor health at age 16 does not influence results (model 4).

When interaction terms for pairings of independent variables are all included in the analysis none appears to be significant. However, sex-disaggregating the data (Tables 3 and 4) exposes different associations between the various social determinants and health outcomes for men and women. Financial strain and low education, the proxy measures of SES, are significantly related to poorer health outcomes among women but not men. For men the only precursor of poorer health is perceived gender inequity.

**Table 3:** Logistic regression analyses for self-rated health at age 42. WOMEN.

	Bivariate		Multivariate^a^	
	OR	CI	OR	CI
Lack of financial strain	0.94	0.90–0.98	0.95	0.91–0.99
Gender inequity	1.09	0.88–1.37	1.02	0.81–1.30
Low education	1.86	1.20–2.90	1.71	1.08–2.67

a after control for reversed causation.

**Table 4:** Logistic regression analyses for poor self-rated health at age 42. MEN.

	Bivariate		Multivariate^a^	
	OR	CI	OR	CI
Lack of financial strain	0.93	0.88–0.98	0.94	0.89–1.01
Gender inequity	1.34	1.03–1.75	1.34	1.02–1.75
Low education	1.19	0.74–1.90	1.23	0.73–2.02

a after control for reversed causation.

## Discussion

We do not know the direction of the perceived gender inequity measured, that is, whether respondents held positions of power or powerlessness relative to their partners. It is tempting to expect that social norms prevail and men hold power when inequities are identified, however this would be an assumption. Our findings speak only to the lack of association for women and statistically significant association for men between living within a relationship where power imbalances exist and self-reported health, and not to the individual health effects that might arise from position within that imbalance. Therefore, although the question regarding gender inequity did not specify the direction of that inequity, our analyses identify that there are sex specific aspects to the health effects of perceived gender inequity at home. The self-rated health of those women studied appeared to be somewhat insulated from harm arising from domestic inequities, whereas men's health suffered when inequity existed. Our findings replicate others showing that in a variety of settings masculine behaviour by either women or men may decrease and detract from the ability to neutralize deleterious external inputs.[20]

Sweden is ranked as the most gender equal country in the world, a macro-level characteristic that may have multilevel effects including an impact on individual health.[21] Never-the-less, at the individual level there is research by Rothstein suggesting that Swedish women continue to take greater responsibility for domestic work and childcare and that this may affect their roles and positions in the workforce.[22] This also suggests, although does not ascertain, that women may be in positions of disadvantage when describing perceived gender inequities in the current study. Our finding, that a perception of domestic gender inequity is more frequently reported by women, is in keeping with Rothstein's research, although a relative lack of financial strain amongst females may mean that domestic inequity does not translate into workplace disadvantage.

Across the relatively homogeneous population studied we have identified some sex differences (see Table 1). Men had significantly lower educational attainment than did women (p = 0.005) but were less likely to suffer from financial strain (p = 0.001). This could suggest that in this setting financial strain is more closely aligned with the combined educational status of the household than each individual within it or that male occupational remuneration is less linked to education level than is the case for women.

When men and women are considered together (Table 2, model 4), higher education and lack of financial deprivation are directly associated with self-reported health while sex and perceived gender equity are not. The grouping of results for both sexes obscures significant sex differences and illustrates the importance of sex-disaggregating data. Including all interaction terms in the regression, in an attempt to identify effect modification of sex on the relationship between the other independent variables and health outcome also yields no statistically significant findings. It is only when data are sex-disaggregated and reanalyzed separately for women and men (Tables 3 and 4) that sex specific relationships between each of education, financial deprivation, perceived gender inequity and health emerge.

In contrast to existing research our findings indicate that individual education level is directly associated with health for women but is not significant for men. The association for women is strong enough that in the collective model (Table 2, model 4) it masks a lack of significance among males (as seen in Table 4). There are a variety of possible explanations for our findings. The equalizing effect of social programs in Sweden may correct for economic disadvantage of lower education for both sexes. Incomes derived from traditional male blue collar jobs may exceed those received by women of the same class and education level. Women, overall, had higher educational attainment than did men so that in contrast to findings in more traditional societies, household educational status that accounts for a family income benefit derived from having a female partner with higher education may more accurately represent lived SES among men than among women. Household education level is unavailable, therefore it is not possible to test whether in the current Swedish context of egalitarianism a woman's educational level may have some bearing on the SES of her partner while his educational attainment has no effect on her SES.[7], [8]

Lack of financial strain also appears to confer health benefits in the aggregated, multivariate model (Table 2, model 4) however disaggregation shows this benefit is only significant for women. Again, the association between financial deprivation and poorer health, overall, may speak to the equalizing influence of Sweden's social programs that do not correct income imbalances but minimize their effects. The sex difference in effect may be the outcome of a partial reversal of traditional sex roles and gendered opportunities in this egalitarian environment.

To the best of our knowledge ours is the first study to include an explicit indicator of perceived gender inequity in one's relationship, and consider whether it changes the association between SES and self-reported health. On the whole, a significantly greater proportion of women reported gender inequity (p = 0.007). When considered alone, gender inequity predicted poorer health outcomes overall (Table 2, model 0), however this association disappeared after adding financial deprivation, sex, and education to the model. Once again, the lack of observed effect at the collective level masked a sex difference revealed when the data were sex-disaggregated. Gender inequity was predictive of poorer health in men independent of measures of SES, but was of no predictive value in women.

In contrast to most existing findings, we have shown that for men, characteristics of the home environment had an impact on general health whereas socioeconomic measures did not, and that the reverse was true for women. While somewhat counter-intuitive, these findings may reflect the egalitarian nature of Swedish society and a loosening of rigid and traditional sex roles in parenting and the workplace. Backhans has shown that when Swedish women move into conventionally male occupational roles their longevity advantage diminishes.[23] Perhaps a similar reversal of fortune explains our data; as women's options expand to include those historically restricted to men, the social determinants of female health make a similar shift toward those previously associated with men. Conversely, as men take on more female roles such as parenting, the inputs that shape their health may align more closely with those traditionally associated with being female.[24] Our findings are consistent with Cooper's [3] suggestion that greater acceptance of gender inequity at the group level may have adverse health effects for women. In the Swedish environment where gender equality is valued, this cultural norm may offset or negate the deleterious health effects of individual gender inequities for women.

A recent study, also using the Northern Swedish Cohort, identified a direct association, greater for men than women, between perceived gender inequity and psychological distress.[25] While we found that women's self-reported health is not harmed by gender inequity, a sense of unfairness within one's relationship may have psychological costs for both women and men, costs that may translate into a perception of poorer physical health among men but do not undermine women's sense of physical wellbeing.

Our research has some limitations. The population studied is relatively homogeneous, limiting generalizeability while conferring robustness since confounding factors such as differences in religion, culture, or access to social programs are not operative. The meaning of deprivation or financial strain is, necessarily, contextual, and not absolute. For example, inability to go to the cinema would not have universal meaning but was part of the composite measure used in this research. However, deprivation is, by nature a relative measure. It is relative deprivation rather than absolute income that seems most meaningful in existing research and hence we have chosen it as a measure of SES. It is also a measure that has been shown to have meaning for men and women. As discussed earlier, our measure of gender inequity was subjective and did not identify the direction of that inequity. Lastly, there may well be unmeasured characteristics such as occupation, health behaviours, aspects of resiliency, etc, that explain some of the observed differences in self-reported health.

In Sweden there is a relatively high degree of familiarity with, and acceptance of the value of gender equality. As a result, direct questioning about gender fairness in one's home environment is feasible. The large proportion who responded to this question among the Northern Swedish Cohort likely implies that participants understood the meaning of domestic gender equity. Those few existing studies that have examined whether gender equity is associated with health have relied on proxy measures of equity such as sex differences in self-reported time spent on housework or parenting. The choice of which measures to use in future research should be informed by a realistic assessment of participants' fluency with concepts of gender and equity and will, therefore vary from country to country. Our aim was not to define an absolute measure of a gender equal or unequal relationship, but rather to look at whether individuals' perceptions of inequities had some bearing on perceptions of health. Our findings do suggest the importance of considering self-reported inequities within the home environment as explanatory factors for physical wellbeing.

This is a first study of the general health effect of domestic gender inequity considered in conjunction with SES (as measured by relative financial deprivation and education level). While higher education and less financial strain predict better self-reported health among women the pattern for men is noticeably different. Only gender inequity in one's primary relationship is associated with poorer male health. Gender inequity at the individual level has less impact on health than the “wounds” caused by financial deprivation among women, but not men. The gender difference is interesting, needs greater exploration, is somewhat in keeping with multilevel studies of less gender equal societies cited earlier and showing that men have poorer mental health outcomes than women, but is counterintuitive. It would, never-the-less, appear that in a society that values equality, aspects of female gender roles increase immunity to, while being male diminishes resilience in the face of gender inequities in one's primary relationship. It would also appear that the associations between gender and health are only revealed when data are sex-disaggregated.

## Supporting Information

Appendix S1 Relevant survey questions (Northern Swedish Cohort Survey)
(DOC)
