# Supplementary material for: Relative Health Effects of Education, Socioeconomic Status and Domestic Gender Inequity in Sweden: A Cohort Study
Source: PLoS One. 2011 Jun 29;6(6):e21722. doi: 10.1371/journal.pone.0021722 (PMC3126845; doi:10.1371/journal.pone.0021722)
Supplement: Appendix S1 — Relevant survey questions (Northern Swedish Cohort Survey) (DOC) [file pone.0021722.s001.doc]

APPENDIX S1

Relevant questions from the Northern Swedish Cohort Survey (translated from Swedish)

*Age 42: Which is your highest education?*

’Compulsory School’/

’Two-year upper secondary education’/

’Three-four upper secondary education’/

‘Academic degree’/

’Post-secondary education’/

*Age 42: How do you consider your health status in general?*

Answer alternatives: “Good”, “Poor”, “Something in between good and poor”.

*Age 42: Are you cohabiting or married?*

Answer alternatives: “Yes”, “No”

*Age 42: Have you been forced to do without any of the following during the last twelve months:* Cooked meal

Buying clothes they or the family needed

Paying bills on time

Going to the cinema/concert/theatre

Inviting friends home

Travelling to see relatives or friends

Buying presents

Going on vacation

Subscribing to a newspaper

Spending time on hobbies or leisure activities

Going to restaurants/pubs

Answer alternatives: “often”, “seldom”, “never”, “non applicable”.

*Age 42: How gender equal do you consider your couple relationship to be*

Answer alternatives: “totally gender equal”, “quite gender equal”, “somewhat gender equal”, “not especially gender equal”, “not gender equal at all”.

*Age 16:*

*How often have you had nervous problems during the last 12 months?*

*How often have you felt sad during the last 12 months?*

Answer alternatives “Never”, “Sometimes” “Often” “All the time”

*Age 16: Have you had the following symptoms during the last 12 months?*

‘Headache, migraine’/

‘Cold’/

‘Cough’/

‘Gastritis, ulcer’/

‘Other stomach ache’/

‘Nausea’/

‘Weightloss’/

‘Diarrhoea’/

‘Constipation’/

‘Lack of appetite’/

‘Shoulder pain’/

‘Pain in extremity’/

‘Backache, hip pain, sciatica’/

‘Asthma’/

‘Fatigue’/

‘Breathlessness’/

‘Dizziness’/

‘Overstrain’/

‘Eczema’/

‘Rash, psoriasis’/

‘Allergy’/

Answer alternatives “No problems”, “Minor problems” “Serious problems”
